# Targeting chromatin remodeling complexes to treat uveal melanoma

**DOI:** 10.7554/eLife.109262

**Published:** 2025-09-30

**Authors:** Jonas A Nilsson

**Affiliations:** 1 Sahlgrenska Center for Cancer Research, Institute of Clinical Sciences, Sahlgrenska Academy, University of Gothenburg Gothenburg Sweden

**Keywords:** BAF, chromatin remodeling, uveal melanoma, SOX10, SMARCA4/SMARCA2, Mouse

## Abstract

A novel compound that inhibits the BAF chromatin remodeling complex causes regression in an animal model of the incurable cancer uveal melanoma.

**Related research article** Centore RC, Soares LMM, Topal S et al., 2024. Pharmacologic inhibition of BAF chromatin remodeling complexes as a therapeutic approach to transcription factor‑dependent cancers. *eLife* **13**:RP93478. doi: 10.7554/eLife.93478.

Uveal melanoma is a type of cancer in a region of the eye called the uvea. It is typically treated with radiation, which cures about half of patients. However, half of the patients subsequently develop metastases – predominantly to the liver – which remain largely incurable.

A predisposition to metastasis is strongly associated with tumor genetics (Robertson et al., 2017). In particular, a gene called *BAP1* codes for an enzyme that has a role in suppressing tumor metastasis, and the loss of this gene correlates with aggressive disease. BAP1 regulates the transcription of certain genes, but the key downstream genes and non-enzymatic functions of BAP1 that are involved in the suppression of metastasis are not fully understood.

Approved systemic treatments for metastatic uveal melanoma include a T-cell engager called tebentafusp, and hepatic perfusion with a drug called melphalan (Huibers et al., 2025). Although these approaches have extended survival beyond two years in some subsets of patients, progression of the cancer is common. Other approaches include cell therapies – such as tumor-infiltrating lymphocytes and CAR-T cells – but these have not yet been approved for public use. Consequently, there is a pressing need for new approaches to the treatment of uveal melanoma.

One promising approach involves harnessing the fact that gene transcription is rewired in cancer cells. Several strategies are available for doing this, including drugs that act on transcription factors, RNA polymerases and various enzymes involved in the modification of histones. In uveal melanoma, early-phase clinical trials have explored a number of these strategies, including drugs that inhibit DNA methyltransferase, the BET bromodomain family of proteins, and histone deacetylases (Wei et al., 2022). The results of these trials have been mixed to date, although a number of studies are still ongoing.

Another approach is to target protein complexes called chromatin remodeling complexes. These complexes use energy from ATP hydrolysis to reposition, eject or exchange nucleosomes, and thereby regulate DNA accessibility for transcription, replication and DNA repair (Figure 1). Chromatin remodeling complexes are frequently mutated or dysregulated in cancer, which makes them a promising target for drug treatments. Many inhibitors are in preclinical testing, but comparatively few have advanced into the clinic, which makes two recent papers by researchers at Foghorn Therapeutics in the US particularly relevant.

**Figure 1.**
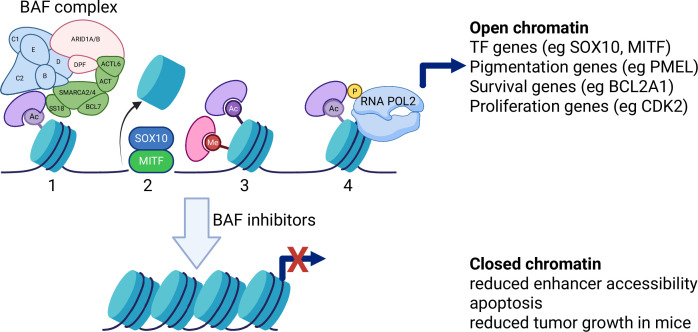
Inhibiting the BAF complex. The BAF complex (top left) contains many different subunits, including a number of different ATPases (SMARCA2/4). When this complex binds to chromatin, a number of different processes can take place: nucleosomes (blue disks) can be ejected (1); the genes in the DNA (black line) become more accessible (2); various epigenetic writers and readers (Me and Ac) can be recruited (3); RNA polymerase II can be recruited (and phosphorylated) in order to start the transcription of a wide range of genes. These include transcription factor genes (such as SOX10 and MIFT), pigmentation genes, survival genes and proliferation genes. BAF inhibitors such as FHD286, FHT1015 and FHT2344 prevent the BAF complex binding to the chromatin, which adopts a closed conformation (bottom). This stops the transcription of the genes, causing uveal melanoma cells to undergo apoptosis, which leads to reduced tumor growth in mice. Created in BioRender.

In one of these papers, published in the Journal of Medicinal Chemistry, Rishi Vaswani, Kevin Wilson and colleagues report that a compound called FHD286 can inhibit both of the ATPase subunits in a chromatin remodeling complex called BAF (Vaswani et al., 2025). FHD286, which can be taken orally, has demonstrated antitumor activity in mouse models of uveal melanoma and acute myeloid leukemia, and is currently being evaluated in phase 1 clinical trials.

In the other paper, in eLife, Richard Centore, Luis Soares, Salih Topal and colleagues report the discovery of two new compounds, FHT1015 and FHT2344, that can also inhibit both of the ATPase subunits in BAF (Centore et al., 2024). The compounds were shown to have structures and modes of action that were different to those of previously known dual inhibitors, and to have little impact on other ATPases. In *in vitro* experiments, Centore et al. showed that the inhibitors caused changes in chromatin accessibility at the binding sites of a number of key transcription factors across a range of cancer cell lines. The researchers then showed that FHT2344 was tolerated in a mouse model of uveal melanoma and caused dose-dependent tumor regression.

Mechanistically, the inhibitors selectively reduce chromatin accessibility at promoter-distal enhancers – particularly super-enhancers – co-occupied by three transcription factors: SOX10, MITF, and TFAP2A (Figure 1). Short-term treatment decreases the occupancy of these sites, without an immediate drop in protein levels: the transcription of SOX10 falls first, followed by MITF. These events correlate with a broader transcriptional shutdown and apoptosis. *In vivo*, the levels of SOX10 (and a protein called FGF9) decline in tumors in line with exposure to the inhibitors, providing means to assess target engagement in clinical samples.

Access to well-characterized BAF inhibitors will be valuable for discovering rational combinations of drugs for the treatment of uveal melanoma. However, it will be important to vet these combinations in animal models to make sure they do not block the immune system’s important role in surveilling cancer cells. This can be done in mice carrying mouse tumors, or in mice where both human tumors and immune cells known as T lymphocytes are simultaneously grafted (Karlsson et al., 2024).
